# Are Vegan Alternatives to Meat Products Healthy? A Study on Nutrients and Main Ingredients of Products Commercialized in Brazil

**DOI:** 10.3389/fpubh.2022.900598

**Published:** 2022-05-27

**Authors:** Bernardo Romão, Raquel Braz Assunção Botelho, Eduardo Yoshio Nakano, António Raposo, Heesup Han, Alejandro Vega-Muñoz, Antonio Ariza-Montes, Renata Puppin Zandonadi

**Affiliations:** ^1^Department of Nutrition, University of Brasilia, Brasília, Brazil; ^2^Department of Statistics, University of Brasilia, Brasília, Brazil; ^3^CBIOS (Research Center for Biosciences and Health Technologies), Universidade Lusófona de Humanidades e Tecnologias, Lisboa, Portugal; ^4^College of Hospitality and Tourism Management, Sejong University, Seoul, South Korea; ^5^Public Policy Observatory, Universidad Autónoma de Chile, Santiago, Chile; ^6^Social Matters Research Group, Universidad Loyola Andalucía, Córdoba, Spain

**Keywords:** meat substitutes, plant-based, meat, label, nutritional composition, ingredients

## Abstract

Proteins are essential components in human nutrition, and animal products are usually the primary sources of human ingestion. However, the number of adherents to vegetarian and vegan diets has grown significantly, highlighting the need for alternatives to replace animal proteins. Meat substitutes aim to mimic the nutritional value and sensory characteristics of meat. However, studies suggest differences in their composition. This study is the first to evaluate Brazilian meat substitutes' nutritional quality and ingredients. A quantitative cross-sectional survey was performed in three steps: (i) Sample mapping of products commercialized nationwide; (ii) Ingredients and nutritional data collection and classification; (iii) Statistical analysis. One hundred twenty-five meat substitutes were included and described. The primary protein sources were soy, gluten, and pea protein ingredients. Vegan meat substitutes presented similar energy and protein values, with few exceptions among samples, with vegan canned fish alternatives presenting less protein than their counterparts. Overall vegan products did not differ regarding sodium levels but showed high amounts to compose a lunch or dinner meal. Vegan meat substitutes showed higher carbohydrates, dietary fiber concentrations, and few differences regarding total and saturated fat. Vegan meat substitutes may contribute to the adherence and maintenance of vegan and vegetarian diets. However, future studies about the implemented ingredients are needed.

## Introduction

Proteins are an essential dietary component contributing to building muscle fibers and the immune system and sustaining many vital functions. Animal-based products are one of the primary sources of human protein ingestion (1). Since ancient times, meat has been an essential component of the human diet due to its nutritional aspects (proteins of high biological value, iron, and vitamin B12), sensory characteristics, and cultural aspects (2–4). Even though the type and amount of ingested meat differ among populations and cultures, most Western countries' main meals include meat combined with vegetables (2). The per capita global meat intake is projected to be 35 kg/year (5). Given the expected population growth and increased influx of low and middle-income countries, the global demand for animal-based products would overcome the world's capacity up to 2,050 (6). On the other hand, people are concerned about the effects of meat-eating on their health and the environment since plant-based diets have been pointed out as one measure to face climate change and non-communicable diseases (5, 7). Animal-based meals require more environmental resources (e.g., land use and freshwater) than plant-based meals. Therefore, adherence to plant-based diets is growing worldwide, mainly for health, ethical, cultural, and environmental reasons (8).

The term plant-based diet can be related to either vegetarian and vegan diets (9–12) or diets that are mostly (but not necessarily exclusively) based on plant foods (13–15). There is no data on the prevalence of plant-based diets adoption worldwide. However, vegetarianism has acquired a lot of attention and supporters, especially in Asia (19% of the population is vegetarian). Vegetarianism is most common in Africa and the Middle East (16%), followed by 8% in South and Central America and 6% in North America. Europe has the lowest prevalence, with only 5% of the population being vegetarian (16). Between 2012 and 2018, the number of vegetarians in Brazil climbed from 8 to 14% (17). Consumer interest in lowering meat consumption and opting for plant-based cuisine has sparked food industry innovations to capitalize on this trend (18).

It is essential to highlight that the acceptance of plant-based meat substitutes is related to several factors, including cost, familiarity, psychological, environmental, and cultural factors (19). The food industries tend to produce plant-based alternatives similarly to their animal-based counterparts, mainly considering physical and sensory aspects (taste, texture, visual appearance, and cooking method). They include several ingredients, such as products based on legumes, grains, nuts, fungi, and additives, such as antioxidants and thickeners (8, 18–20). However, studies suggest that plant-based meat substitutes lack nutritional quality being low in protein and rich in sodium, fat, calories, fiber, and total carbohydrates, but few studies analyzed the meat-substitute products commercialized countrywide (8, 18–20). To our knowledge, there are two investigations on the nutritional profile of substitute beef burgers in the USA and Australia (21, 22). However, none was performed in Brazil, analyzing the nutritional composition of plant-based meat substitutes. Given the expansion of plant-based meat to a broader population, the potential consequences of its consumption for public health need to be addressed based on the knowledge of the nutritional profile of plant-based meat substitutes. It is fundamental to provide adequate dietary choices and visualize potential nutritional differences between meat substitutes and their animal-protein counterparts. The central hypothesis of this study is that vegan products commercialized in Brazil do not resemble their animal counterparts regarding nutritional aspects. The secondary hypothesis is that vegan products present more carbohydrates, sodium, and lower protein than their animal counterparts. Therefore, our study aimed to evaluate the nutritional quality and the main ingredients used in vegan meat analogs commercialized in Brazil.

## Materials and Methods

This comparative cross-sectional quantitative study was conducted in three steps: (i) Sample mapping; (ii) Data collection and classification; (iii) Statistical analysis, as described below.

### Sample Mapping

The inclusion criteria for the meat substitutes sample in the study were: (i) the presence of the seal “Vegan Product,” offered by the Brazilian Vegetarian Society (SVB^®^); (ii) products commercialized in hyper and supermarket chains present in the five Brazilian regions and/or food stores with national and regional coverage. The exclusion criteria were fresh foods or other vegan products whose objective is not to mimic any meat-based counterpart based on animal protein. This study did not include vegan and regular meat products labeled with the nutritional claims “low fat” and “low salt,” since they could have biased the results. E-commerce was consulted through search platforms (Google^®^), Brazilian online vegan products resellers, and on social media (Instagram^®^, Facebook^®^, and Twitter^®^), through hashtags and nominal searches to achieve national coverage of the meat substitutes sold in the Brazilian market. The investigation was conducted from February 1st, 2021, to December 1st, 2021.

The search was conducted in 3 phases: a researcher searched for the vegan products in the first phase. Then, a second researcher repeated the search process and analyzed the need to include more products. As a result, two independent academics double-checked the precision of the extracted data. Finally, a third coordinating researcher critically analyzed the data, determining the final sample based on the inclusion criteria.

Then, the meat substitutes were classified into ten categories as their meat product counterpart: *Hamburgers; Minced Beef; Meatballs; Breaded Chicken; Chicken Hamburgers; Chicken Breast; Canned fish; Fish Cakes; Sausages and Hams*.

In addition, for comparison purposes, three samples of three different Brazilian best-selling animal products to each meat product counterpart were also included in the study.

### Data Collection

Data collection followed previous studies on modified versions of commercialized products (6, 23–25). Products from the most significant producers were chosen, covering most of the Brazilian market. The qualitative and quantitative data reported on the products were recorded, including firm name, brand name, descriptive name, ingredient's list, nutrient information, and serving size. Information about the ingredients and nutrient values was collected from the food labels. They allow consumers to choose healthy and adequate foods according to their dietary pattern by the nutrient profile and ingredients' list. According to the Brazilian legislation, it is mandatory to describe the serving size (g), energy value (kcal), carbohydrates (g), added sugars (g), proteins (g), fats (g), saturated fats (g), dietary fiber (g) and sodium (mg) (26). Therefore, we used these parameters to compare the products from the sample mapping phase. For standardizing and comparison purposes, all values were converted to the serving size of 100 g. To prevent the double inclusion of products, if more than one product had the same composition, they were only considered once.

### Statistical Analysis

Data regarding the included samples' energy value (kcal), carbohydrates (g), added sugars (g), proteins (g), fats (g), saturated fats (g), dietary fiber (g), and sodium (mg) were calculated on their respective means ± Standard Deviations (SD). A comparison between nutritional values of meat substitutes and their respective animal protein-based products was carried out with a non-parametric Mann-Whitney test with a confidence level of 95% (*p* <0.05). Two-tailed hypotheses were considered in the test. *Microsoft Excel*^®^ *(USA*, 2021) and *SPSS*^®^ *version 22.0 (IBM SPSS Statistics, Version 22.0, IBM corp., Chicago, IL USA*, 2020) were used to perform the tests.

For graphical visualization, a word cloud was generated with the implemented ingredients of vegan meal analogs, given that higher frequencies are represented with more prominent words in the cloud (*Wordclouds*^®^*, 2022*) (27). For the word cloud generation, protein sources were grouped according to their main matrix; for example, texturized soy protein, isolated soy protein, and soybeans were all grouped as “soy.” Furthermore, information regarding the ingredients was represented by percentages in a heatmap where the color indicates the ingredient's presence according to the stipulated categories. *GraphPad Prism*^®^ *(San Diego, CA, USA, 2022)* was used to generate the heatmaps.

## Results

The total amount of 125 products were included for evaluation. Most of them was red-meat product analogue (*n* = 62; 49.6%), 25.6% (*n* = 32) were chicken-meat product analogues, 14.4% (*n* = 18) were pork-meat product analogues and 10.4% (*n* = 13) were fish-meat product analogues. Among the types of meat substitutes, 28% (*n* = 35) were classified as *Hamburgers*, 7.2% (*n* = 9) as *Minced Beef*, 14.4% (*n* = 18) as *Meatballs*, 11.2% (*n* = 14) as *Breaded Chicken*, 4.8% (*n* = 6) as *Chicken Hamburgers*, 9.6% (*n* = 12) as *Chicken Breast*, 6.4% (*n* = 8) as *Canned fish*, 4% (*n* = *5)* as *Fish Cakes*, 9.6% (*n* = 12) as *Sausages* and 4.8% (*n* = 6) as *Hams*. Table 1 shows the vegan and animal samples' energy value (kcal), carbohydrates (g), proteins (g), fats (g), saturated fats (g), dietary fiber (g) and sodium (mg) by means and standard deviations (SD). Complete information regarding nutritional value, ingredient list and serving size in all included samples is available at the Supplementary Tables 1, 2.

**Table 1:** Means and standard deviations of the nutritional values per 100 g of serving of the included samples.

	**Energy (Kcal)**			**Carbohydrates (g)**			**Protein (g)**			**Total Fat (g)**			**Saturated Fat (g)**			**Dietary Fiber (g)**			**Sodium (mg)**		
**Category**	**Vegan**	**Animal**	***p***	**Vegan**	**Animal**	***p***	**Vegan**	**Animal**	***p***	**Vegan**	**Animal**	***p***	**Vegan**	**Animal**	***p***	**Vegan**	**Animal**	***P***	**Vegan**	**Animal**	***p***
Hamburgers	216.18 ± 77.61	228.33 ± 46.54	0.644	18.22 ± 12.95	3.13 ± 0.57	**0.002**	14.77 ± 9.34	16.67 ± 1.91	0.168	8.91 ± 6.63	16.88 ± 4.38	**0.048**	3.20 ± 4.19	6.21 ± 1.38	0.056	5.60 ± 4.53	0.54 ± 0.94	**0.029**	434.49 ± 185.90	595.00 ± 77.59	0.074
Minced beef^*^	192.81 ± 29.73	247.67 ± 6.51	**0.009**	12.91 ± 12.14	0 ± 0	**0.012**	14.25 ± 5.95	23.00 ± 1.00	0.064	10.00 ± 3.37	15.67 ± 2.08	**0.009**	3.01 ± 3.51	6.67 ± 1.15	0.064	5.77 ± 4.61	0 ± 0	**0.012**	572.96 ± 159.98	76.00 ± 4.36	**0.009**
Meatballs	156.18 ± 76.06	200.00 ± 43.37	0.307	7.36 ± 6.84	7.58 ± 3.87	0.740	17.78 ± 12.67	13.17 ± 1.61	0.740	5.55 ± 5.45	11.04 ± 3.52	**0.010**	1.20 ± 2.71	5.29 ± 1.8	**0.017**	5.58 ± 5.72	0.42 ± 0.36	**0.047**	451.60 ± 212.22	649.17 ± 85.49	0.125
Breaded chicken	216.12 ± 62.28	220.51 ± 35.43	0.953	17.39 ± 9.80	15.13 ± 2.91	0.768	12.97 ± 2.76	13.33 ± 1.94	0.768	10.7 ± 6.37	11.79 ± 3.87	0.591	1.28 ± 1.25	4.08 ± 1.06	**0.021**	4.32 ± 1.85	1.56 ± 0.24	0.068	499.62 ± 194.57	489.49 ± 128.34	1,000
Chicken hamburgers	201.92 ± 67.97	202.92 ± 43.68	1,000	10.07 ± 6.81	3.08 ± 0.14	0.167	18.25 ± 15.87	16.67 ± 1.91	0.262	9.29 ± 5.87	13.42 ± 3.47	0.381	5.04 ± 5.45	4.17 ± 1.39	0.905	6.08 ± 7.13	0.75 ± 0.66	0.095	372.78 ± 190.54	546.25 ± 245.95	0.381
Chicken breast^*^	173.63 ± 67.62	163.33 ± 2.89	1,000	8.90 ± 5.70	0 ± 0	**0.009**	21.77 ± 14.00	30.67 ± 0.58	0.101	5.17 ± 4.96	3.53 ± 0.12	0.734	0.63 ± 0.66	1.00 ± 0	0.557	6.79 ± 6.27	0 ± 0	**0.009**	458.67 ± 244.82	74.00 ± 0	0.081
Canned fish (tuna and sardines)	194.22 ± 87.77	159.33 ± 28.04	0.630	14.70 ± 5.68	0 ± 0	**0.013**	8.75 ± 4.64	24.89 ± 2.99	**0.012**	11.00 ± 7.07	6.73 ± 4.08	0.630	2.63 ± 1.56	2.10 ± 1.23	0.630	6.41 ± 5.58	0 ± 0	**0.013**	416.02 ± 186.91	415.44 ± 133.51	0.921
Fish cakes	193.70 ± 60.85	164.25 ± 44.95	0.571	13.83 ± 9.24	9.56 ± 9.45	0.786	10.07 ± 3.99	16.24 ± 12.18	1,000	11.75 ± 6.85	8.72 ± 1.97	0.393	1.53 ± 1.11	2.09 ± 1.49	0.571	4.67 ± 2.77	2.59 ± 4.01	0.25	482.03 ± 60.89	221.27 ± 157.05	**0.036**
Sausages	189.69 ± 47.55	272.67 ± 44.38	**0.031**	8.63 ± 6.05	2.67 ± 0.95	**0.018**	14.20 ± 3.46	14.93 ± 2.31	0.536	9.85 ± 4.40	22.33 ± 5.51	**0.009**	1.73 ± 2.00	7.47 ± 1.50	**0.018**	4.71 ± 5.08	0 ± 0	**0.008**	572.35 ± 119.33	1122.67 ± 326.01	**0.009**
Hams	251.97 ± 121.17	159.17 ± 124.13	0.262	14.57 ± 18.83	3.08 ± 4.11	0.167	19.64 ± 12.85	14.42 ± 2.13	0.167	12.83 ± 7.49	9.75 ± 12.99	0.381	0.99 ± 0.52	3.33 ± 4.47	0.905	5.61 ± 3.09	0 ± 0	**0.043**	954.03 ± 300.41	1065.83 ± 417.41	0.714

*It is considered significantly different when p < 0.05, highlighted in bold numbers;*

* *Samples of raw and unseasoned meat (animal) were considered*.

In the vegan products, *hams* presented the highest energy concentration (251.97 ± 127.17 kcal/100 g), and *meatballs* offered the lowest (156.18 ± 76.06 kcal/100 g). Among the animal products, *sausages* were classified with the highest energy values (272.67 ± 44.38 kcal/100 g) among all samples, and the *canned fish*, had the lowest (159.33 ± 28.04).

Significant differences (*p* < 0.05) were found in *minced beef* and *sausages* comparing vegan and animal products, with the animal protein-based versions presenting the highest values **(**192.81 kcal ± 29.73/100 g) and (189.69 kcal ± 47.55/100 g).

Statistical analysis showed significant differences between vegan and animal versions of hamburgers, minced beef, chicken breast, canned fish, and sausages regarding the carbohydrate content. In these samples, vegan products presented higher values for carbohydrates than their animal counterparts. The *hamburger* (18.22g ± 12.95/100 g) was the category with the highest carbohydrate content among vegan products and breaded chicken among animal products (15.13 ± 2.91).

Between all animal counterparts, *chicken breast, minced beef, and canned fish* did not present carbohydrates in their composition. Vegan *meatballs* presented the lowest values among all vegan products (7.36 g ± 6.84/100 g).

Protein values varied from 8.75 g ± 4.6/100 g (vegan canned fish) to 21.77 g ± 14.00 (vegan chicken breast) among vegan samples. Animal protein *chicken breast* also presented the highest value (30.67 g ± 0.58/100 g) among all animal protein samples. Furthermore, meatballs have the lowest values (13.17 g ± 1.61/100 g) for animal products. Only *canned fish* samples presented statistical differences between vegan and animal counterparts.

Significant differences were found regarding total fat values between animal and vegan versions of *hamburgers, minced beef, meatballs, and sausages. The vegan versions presented* lower values than their animal counterparts. Among the vegan products, *hams* presented the highest value (12.83 g ± 7.49/100 g) for fat, while *chicken breast* (5.17 g ± 4.96/100 g) showed the lowest. Animal-based versions of *sausages* presented 22.33 g ± 5.51/100 g of total fat, the highest value among animal products, and chicken breast, the lowest (3.53 g ± 0.12/100 g). Regarding saturated fat, statistical differences were found between vegan and animal options of *meatballs, breaded chicken, and sausages*, with the animal options presenting higher content than vegan ones. Among vegan options, *chicken hamburgers* showed the highest concentration of saturated fat (5.04 g ± 5.45/100 g), while in the animal counterparts, *sausages* presented the highest values (7.47 g ± 1.50/100 g). *Chicken breast* showed the lowest values for saturated fat in both animal and vegan options, presenting 1.00 g ± 0.00/100 g and 0.63 g ± 0.66/100 g, respectively.

Regarding dietary fiber, vegan *chicken breast* presented the highest values among all samples (6.79 g ± 6.27/100 g), while vegan *breaded chicken* presented the lowest (4.32 g ± 1.85/100 g). Significant differences were found between 70% (*n* = 7) of the vegan options and their animal counterparts, with the vegan options presenting higher fiber content. Animal-based options of *minced beef, chicken breast, canned fish, sausages, and hams* did not show any dietary fiber.

Significant differences were found between vegan and animal counterparts in *minced beef and fish cakes. The vegan versions of these products presented higher concentrations of sodium than* their animal counterparts. *Sausages* also presented statistical differences between animal and vegan options; however, the animal option presented higher sodium values (1,122 g ± 326.01/100 g) than its vegan counterpart (572.35 g ± 119.33/100 g). No differences were found between animal and vegan samples of *hamburgers, chicken breast, canned fish, meatballs, chicken hamburgers, sausages, and hams*.

**Figure 1:**
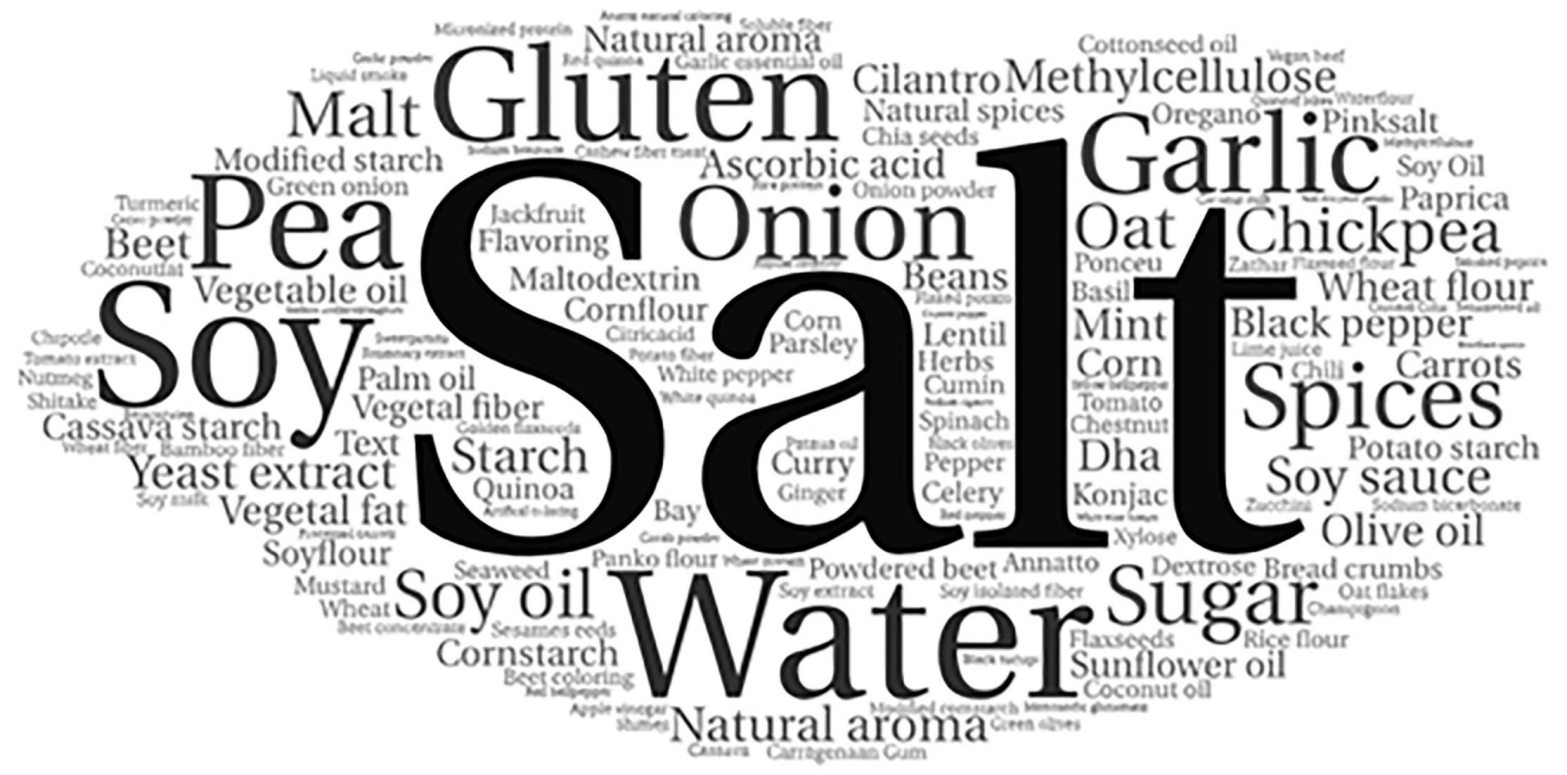
Word cloud generated with the frequencies of implemented ingredients on vegan meat analogs. Higher frequencies were proportionally represented with more prominent graphic representations.

A word cloud with a graphical representation of all implemented ingredients in vegan meat analogs is available in Figure 1. The information regarding the frequency of mentioning the main protein and fat sources in food labels and the food additives in all samples of vegan meat analogs are available in Table 2.

**Table 2:** Main protein and fat sources and food additives according to their frequency of mentioning in all samples' food labels of vegan meat alternatives.

Protein source^*^	***n***	**%**	**Fat source**	***n***	**%**	**Food additive**	***n***	**%**
Protein containing soy products	96	77%	Unspecified vegetal fat	41	32.8%	Methylcellulose	73	58%
Gluten	55	44%	Soy oil	33	26.4%	Natural aroma	39	31.2%
Protein containing pea ingredients	26	20.8%	Sunflower oil	16	12%	Ascorbic acid	23	18.4%
Protein containing chickpea ingredients	20	16%	Olive oil	13	10.4%	Caramel color	13	10.4%
Protein containing beans ingredients	9	7.2%	Cottonseed oil	8	6.4%	Carregenaan gum	7	5.2%
			Palm oil	8	6.4%	Citric acid	3	2.4%
			Coconut oil	5	4%	Guar gum	2	1.6%
			Coconut fat	2	1.6%			
			Palm fat	2	1.6%			

* *Protein sources were grouped according to their main matrix*.

Regarding all utilized ingredients, culinary ingredients such as water, salt, sugar, and spices (including onion, garlic, paprika, smoked paprika, nutritional yeast, and yeast extract) were found in most samples (96%). Soy-based protein-rich products were the most used protein sources (77%), followed by gluten in 44% of the samples.

Ingredients derived from legumes (soy, peas, chickpeas, and beans) are the most used in the studied samples. Different presentations of ingredients based on the same legume were implemented in the vegan meat alternatives. Texturized soy protein, soy protein, and isolated soy protein are soy-based, and texturized pea protein, pea protein, and isolated pea protein are pea-based. Chickpeas and chickpea flour were also used, and beans were used in a lower quantity. It is important to note that all samples combined at least two or more protein sources (Supplementary Table 1). A single sample (0.8%, *n* = 1) utilized cashew fiber as its only protein source.

Unspecified vegetal fat was present in most samples (32.8%); however, while not described in nutritional labels, it was not possible to determine the source of this fat.

**Figure 2:**
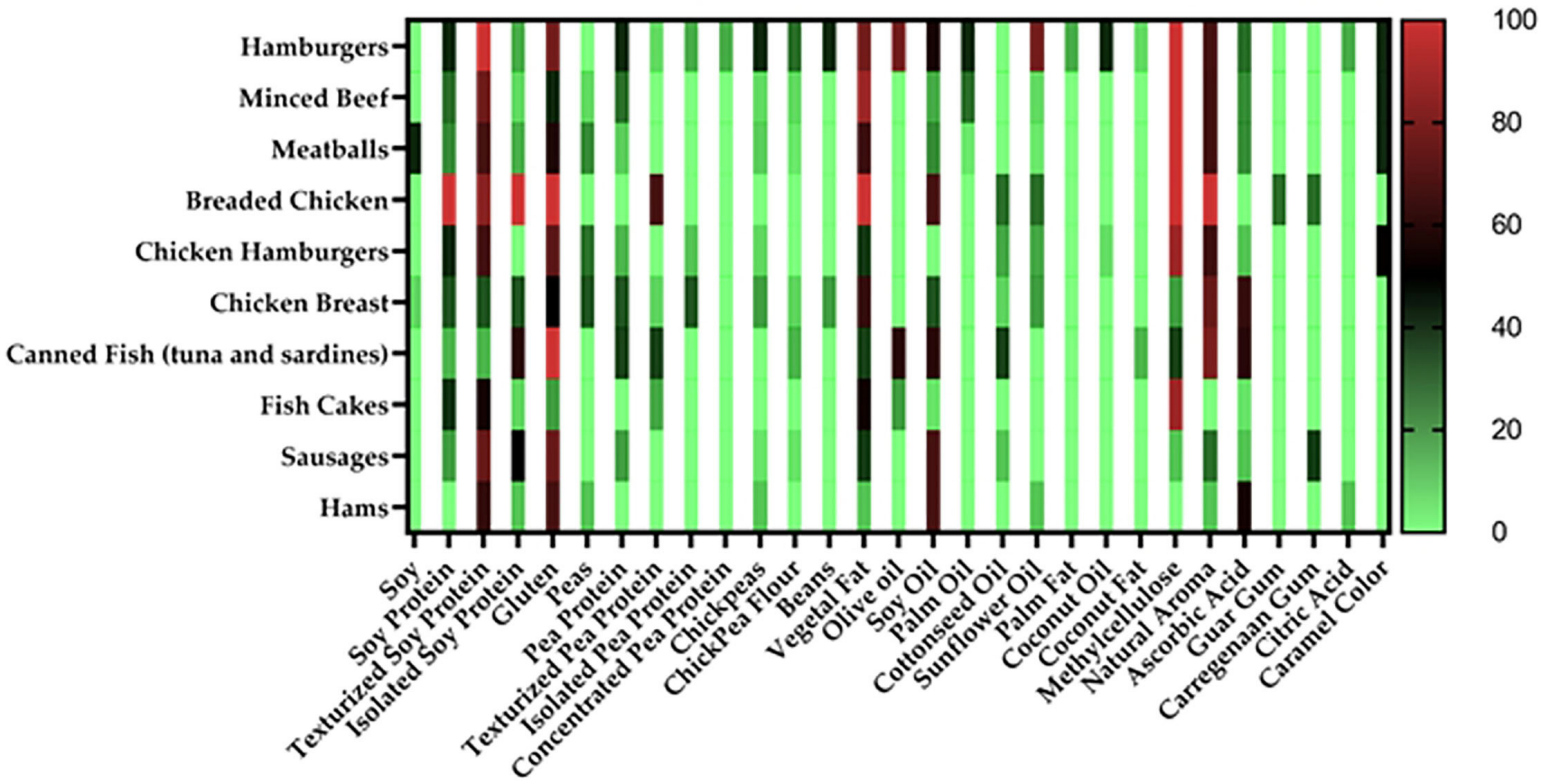
Percentages of frequencies of implemented ingredients in vegan meat alternatives in a heatmap. Colored clusters represent different frequencies according to the scale placed on the right side.

The distribution of the ingredients with frequencies higher than 1.5% among specified categories is described in the heatmap (Figure 2).

In hamburgers, texturized soy protein was present in 100% of the samples, while gluten was in 77.7%. Pea protein was present in only 44% of the samples. Vegetal fat, soy oil, and sunflower oil were used equally among samples (77%). Regarding food additives, methylcellulose was found in 100% of the hamburger samples, a natural aroma in 66%, and caramel color in 44%.

Minced beef samples presented texturized soy protein (77%), gluten (44%), and pea protein (33%) as their primary protein sources. Chickpeas and chickpea flour were found in lower quantities, with a frequency of 11% for both ingredients. Vegetal fat was also predominately used as a fat source (88%), followed by palm oil (33%) and soy oil (22%). In meatballs, soy (as soybeans) was present in 44% of the samples, however, texturized soy protein (67%) and gluten (57%) were present in higher quantities, following the tendency among bovine meat substitutes. Regarding food additives, methylcellulose was also present in 100% of the minced beef and meatballs samples, followed by a natural aroma (66%) and caramel color (44%).

All samples of breaded chicken presented soy protein, isolated soy protein, and gluten. Other protein sources such as texturized protein sources (77%) and texturized pea protein (66%) were also present. As for the implemented fat sources, vegetal fat was found in 100% of the samples, followed by soy oil (77%) and both cottonseed and sunflower oils with 33%. Methylcellulose was present in 100% of breaded chicken samples, similar to bovine meat alternatives. Guar and carrageenan gums were used in 33.3% of breaded chicken samples.

Chicken hamburgers used mainly gluten (72%), texturized soy protein (66%), peas (33%), pea protein (20%), isolated pea protein (12%), and chickpeas (9%) as their protein sources. Vegetal fat was the most used lipid source (44%). Methylcellulose and a natural aroma were the most used food additives, with 88 and 65%, respectively.

A more diverse distribution of protein sources was found on chicken breast alternatives. Gluten (50%), soy protein (37%), texturized soy protein (37%), peas (37%), pea protein (37%), chickpeas (25%), beans (25%), texturized pea protein (17.5%) and chickpea flour (12%) were utilized. Vegetal fat was the most predominant lipid source with 62.5%, followed by soy oil with 37.5% and cottonseed oil with 12.5%. Additives such as natural aroma (72%), ascorbic acid (62.5%), and methylcellulose (25%) were present in this category.

Canned fish presented as protein sources, gluten (100%), isolated soy protein (60%), both texturized and regular pea protein (40%), texturized soy protein (20%) and chickpea flour (20%). Both olive and soy oil were used in 60% of canned fish samples, while vegetal fat and cottonseed oil were present in 40% of the samples of this same category. Coconut fat was found in 20% of the canned fish samples. Considering food additives, a natural aroma was found in 80% of the samples, followed by ascorbic acid (60%) and methylcellulose (40%).

The primary protein source presented in fish cakes were texturized soy protein (55%), soy protein (43%), gluten (25%), texturized pea protein (23%), and isolated soy protein (12%). Similar to the other categories, vegetal fat was the primary lipid source with 54%. Olive oil was the second most used (25%), followed by soy oil (8.33%). Methylcellulose and ascorbic acid were the only additives used, with 88 and 8.33%, respectively.

Gluten and texturized soy protein were present in 75% of the plant-based sausages, while soy and pea protein were used in 25% of the samples. Chickpeas and chickpea flour were used in only 8.33% of the samples. Soy oil was the most used fat source in this category, present in 66.67% of the samples. Vegetal fat was present in 41.67% of the samples, followed by cottonseed oil in 16.67% of the studied products. Carrageenan gum was the most used additive in 41.67% of the samples. A natural aroma was found in 33.33% of the samples, and ascorbic acid and methylcellulose in 16.66% of them.

Gluten was utilized in 66% of hams, followed by texturized soy protein (62%), while chickpeas and peas were found in 16.7% of ham samples. Soy oil was the predominant fat source in hams, present in 66% of the samples. Both vegetal fat and cottonseed oil were used in 16.66% of the samples. Ascorbic acid was the most utilized food additive, present in 55% of the samples. Sixteen percentage of the studied samples used a natural aroma and citric acid.

## Discussion

This study is the first on the nutritional quality of vegan meat substitutes commercialized in Brazil. Plant-based vegan and vegetarian diets are well-known for their association with longevity, higher quality of life, and protection of various non-communicable diseases (28–31). However, the excessive consumption of industrialized foods that substitute animal-based products goes oppositely, and information is scarce regarding this practice within vegetarian and vegan diets (32, 33).

Regarding energy values, despite being a point of attention, vegans and vegetarians tend not to present differences in energy intake compared to omnivorous individuals (28). Therefore, the consumption of industrialized foods might exert a similar influence independently of the adopted diet. A primary concern related to industrialized food consumption is that this product commonly presents a higher energy density and collaborates with an excessive energy intake. It has already been associated with an increased prevalence of diseases related to excessive body mass, such as coronary heart disease and type II diabetes, thus constituting a public health problem (34–36). Brazilian vegan meat substitutes in our study tended to present similar energy values to their animal counterparts. Differences were only found in *minced beef* and *sausages*, different from another study conducted in the United States, wherein general, all vegan meat substitutes presented lower energy values (21).

Given the natural carbohydrate-rich nature of plant-based foods, vegan and vegetarian diets usually present a higher amount of this macronutrient (21, 33, 34). This is possible due to the use of plant-based matrixes, which tend to present fiber in their composition, in contrast with animal-protein meat, which usually does not present this nutrient. Included vegan products presented differences regarding their concentration of carbohydrates in hamburgers, minced beef, chicken breast, canned fish, and sausages, with the vegan options presenting higher values than their animal counterparts.

It is important to highlight that there are diverse carbohydrates in vegetable products, such as starch, polyols, fructose, and galactooligosaccharides. In meat products, starch sources are used due to their properties related to texture improvement, shelf-life extension, cohesiveness, and elasticity, especially in the case of hamburgers, sausages, and hams. These proprieties are mostly associated with starches' capacity to form stable gels through gelatinization (37). In addition, they can also be technological substitutes for fat (38). This tendency is also evident in researched meat substitutes worldwide, where corn and potato starches were implemented (22). Although added sugar is an important carbohydrate present in industrialized preparations (39, 40) and mandatory in labels, according to the Brazilian legislation, the studied meat substitute did not present any added sugar, or no considerable amounts were present in the chosen serving sizes. A major problem involving Brazilian nutritional labels is that nutritional values are described based on the chosen portion by the manufacturer (26), which does not necessarily reflect the usual consumed portion of the product. Thus, nutrients with insignificant amounts (<1%) in the chosen portion are not shown on the nutrition label, and possibly, when adopting the usual amount of consumption, these nutrients would have expressive values that should be described on nutrition labels (41).

Both Protein Intake and Quality Are Important Regarding Vegan and Vegetarian Diets (42). Although it is entirely possible to obtain the proper amount of protein intake in this type of diet, issues related to the nature of the vegetal protein source must be considered. Essential amino acids cannot be synthesized by any endogenous human metabolic pathway (42). Regarding animal protein, usually, a single portion of meat has a satisfactory amount of these amino acids. At the same time, in vegetable sources, one or more sources must be combined to reach the adequate intake (9, 42). Usually, legumes have reduced amounts of methionine, while cereals have a lower lysine concentration. Therefore, combined portions of cereals and legumes should be consumed (42).

Legumes such as soy, chickpeas, peas, and cereals such as wheat usually function as protein sources in meat substitutes. In the included samples, gluten was the second most present protein source. Gluten is a protein complex in cereals such as wheat, rye, and barley that presents unique characteristics on food as adhesion, elasticity, cohesion, and enhance the texture of food products (43). Commonly, its isolated form is sold in the form of vital wheat gluten, an ingredient consisting of isolated starch-free wheat gluten, dehydrated and in flour granulometry (44). This ingredient forms an elastic, tenacious and consistent net whose texture can resemble some types of meat, especially those with a firmer texture, as evidenced in the samples where it was most used, such as hamburgers, breaded chicken, chicken hamburgers, canned fish, sausages, and hams. However, it is important to mention that worldwide there is about 10% of the population (45, 46) following a gluten-free diet (GFD). The high use of gluten in vegan products might limit the consumption of this kind of product by people who need to follow a GFD and opt to follow the vegan dietary pattern.

Soy stood out as the main protein source used in the studied samples, in the forms of texturized soy protein, soy protein, and isolated soy protein. Texturized soy protein is obtained by a thermoplastic extrusion process. Regarding its texture and appearance, this ingredient is sensory similar to animal meat; thus, since its invention, it is already widely used as a meat substitute (47). Isolated soy protein also functions as a texture improver since its proteins behave stably during the cooking process, resulting in firmer products and contributing to coloring by favoring the Maillard reaction (48).

Soybeans characterize one of Brazil's largest production markets, whose profits correspond to a significant portion of the country's gross domestic product (GDP) and move about US$20 billion a year (49, 50). Thus, given its high availability, soybean is widely used in industrialized products, both of animal and plant origin, given its technological characteristics that are considered desirable by the industry (51).

Soy has an aminogram comparable to meat, milk, and egg proteins concerning its nutritional value. From a dietary point of view, it is one of the most used foods to replace ingredients of animal origin (47, 48).

However, one of the main obstacles to soybean consumption is its high allergenic potential. Currently, soy allergy occurs on 0.5% in the general population, with an even higher number in children (about 12%) (52). Thus, one of the emerging alternatives to replace soy in vegan meat substitutes is pea-derived protein. With the same technology used in soy protein derivatives, pea protein has similar nutritional and sensory points of view. It is widely used in products whose goal is to offer soy-free alternatives (53, 54). As evidenced in the study, meat analogs commercialized in Brazil used pea protein in its textured, isolated, and pure form, but in lower quantities compared to soy protein-based ingredients.

Another alternative to protein sources is other legumes, as evidenced by the use of chickpeas and beans. The included samples used only cooked and without processing technology. However, characteristics such as moisture retention, protein concentration, and, consequently, the sensory aspect are impaired without the technological processes employed, for example, thermoplastic extrusion or protein isolation) (47). In this sense, the predominant use of textured versions of proteins derived from legumes was evidenced in the present study instead of unprocessed versions of legumes.

The protein sources used in Brazilian vegan meat analogs are similar to those evidenced in studies conducted in other countries. However, the other studies showed lower protein concentrations than their animal counterparts, thus emphasizing the importance of choosing and combining ingredients for this purpose (21, 22).

Studied vegan versions presented lower values regarding total and saturated fat amounts mainly because vegetables usually present reduced content of these nutrients (9). Fats are essential components in developing processed foods, as their presence is responsible for the high palatability, aftertaste, “crunchiness,” and color commonly associated with this type of product (55). In this sense, given their lower concentration of total and saturated fats and when considering the purposes of vegan meat substitutes, the moderated use of these products in place of their animal counterparts can be beneficial. However, although fats and saturated fats are present in smaller amounts in vegan meat substitutes than their animal counterparts, the presence of these nutrients should be carefully analyzed, as their excessive consumption is intimately linked with the development of non-communicable chronic diseases (27–29, 35, 44)].

Unspecified vegetable fat was the most used source of fat in the studied samples. Although unspecified, the vegetable fat used in industrialized products is usually hydrogenated. This ingredient is obtained from the hydrogenation of vegetable oils, and it adds desirable characteristics from an industrial point of view, such as longer shelf life and greater palatability (38). A characteristic resulting from the hydrogenation of plant oil is its melting point. In the form of vegetable oils, with their reduced melting point, these fat sources remain liquid at room temperature. Hydrogenated vegetable fat, on the contrary, remains solid, thus preserving the sensory characteristics of the food (38). However, it is already well-known that excessive consumption of this type of fat is associated with an increase in the prevalence of chronic non-communicable diseases, thus reinforcing the need for caution regarding its consumption (55, 56). Oils such as soybean, cottonseed, sunflower, and palm were also used in the studied products. Concerning the excessive consumption of these oils, it is noteworthy that these are sources of omega-6 fatty acids, compounds directly related to the production of pro-inflammatory cytokines. Thus, they should then have their consumption moderated (57, 58). Olive oil was used more pronouncedly in the canned fish category, probably because some of these products are commercialized ready for consumption, not requiring cooking and therefore not resulting in physicochemical changes of this oil. However, olive oil tends to present a higher cost; therefore, its use in industrialized products is usually limited.

Dietary fiber is a naturally plant-derived nutrient, and its consumption is associated with weight maintenance and better health of the gut microbiota (40, 59). In technological aspects, the addition of dietary fiber provides favorable characteristics such as greater moisture retention, thus contributing to the texture. However, excessive amounts of fiber can result in more rigid products, increasing chewing, thus constituting sensory impairment (60). All meat substitutes had higher dietary fiber values than their animal-derived counterparts since animal products tend to have low (or not have) fiber. In the last Brazilian survey of risk factors for chronic diseases *(Vigitel)*, the results showed a lower consumption of vegetables and fruits by the Brazilian population, resulting in a lower intake of dietary fiber s can be based on food composition (61). To obtain appropriate intake, the World Health Organization (WHO) recommends consuming at least 400 g of fruits and vegetables (equal to five servings) daily (62). When the recommended daily consumption of five meals was assessed in a research of a Brazilian vegetarian's statewide sample, just 38% of them displayed adequate intake (31) compared to 21% of the general Brazilian population (63). In this sense, vegan meat substitutes could compose Brazilian meals to improve fiber intake among Brazilians (vegetarians or not).

However, it is important to consider that dietary fiber has the physical ability to retain liquids such as water or oil (59, 64). In the context of meat substitutes sold for subsequent cooking, this physical characteristic of dietary fibers can collaborate with greater retention of lipids (when using a cooking method with oil or fat) and, consequently, in higher concentrations of lipids and energy values (64).

The high sodium level in meat substitutes is a trend already evidenced by studies in other countries (21, 22). In the present study, only the vegan versions of *minced beef* and *fish cakes* showed significant differences compared to their animal counterparts, in this case, with higher values. Nevertheless, it is worth noting that the animal version of *minced beef* corresponds to raw fresh food without adding salt, in contrast with the vegan versions that are already commercialized with seasoning. The presence of nitrogenous bases in beef gives it its own accentuated flavor, thus reducing adding salt and other seasonings (64). The animal-based protein has substances in its composition that give its characteristic and accentuated flavor. Besides, its concentration and distribution of amino acids and reducing sugars favor the Maillard reaction, which contributes considerably to the development of these foods (64).

Fish cakes are preparations commonly made with saltwater fish and other seafood. In this sense, the addition of sodium was probably intended to mimic the characteristic flavor of these animal proteins. However, despite a few differences regarding sodium, it is necessary to analyze the contribution of meat substitutes with the recommendation of total daily sodium intake. Currently, WHO recommends a daily value of 2,300 mg of sodium/day (65). Considering lunch in Brazil as a contributor of 40% of the total daily value, a limit of 920 mg of sodium would be established (66). In this sense, 100 g of the included meat substitutes would contribute on average, with sodium values referring to about 30–50% of the lunch. A typical Brazilian food-service lunch comprises a main dish (high in protein—in which products evaluated in this study could be included) and garnishes, rice, beans, and salads, and their respective average contribution regarding sodium values. Therefore, meat substitutes might contribute to an excessive sodium intake since they represent, for some products, almost 50% of the total sodium recommendation for the lunch meal (66).

Food additives are not food compounds but they are used in food products to improve technological and sensory characteristics (67). Methylcellulose was the most used food additive in the studied samples. This ingredient is a hydrocolloid derived from cellulose whose action is directly related to improving the texture and emulsion of phases (68). When used in meat preparations, this ingredient contributes directly to the texture, humidity, agglutination, and integrity of the preparations, already being commonly used in sausages, compacted fillets, hams, and burgers (38). Another hydrocolloid, carrageenan gum, was utilized in sausages and breaded chicken, and it performs similarly to methylcellulose (67, 68). Citric and ascorbic acids are common food additives from the class of preservatives, used to improve of durability and shelf-life by reducing oxidation and water activity (67). A major concern related to artificial food additives is caramel color. This additive is widely used in industrialized preparations and has long been associated with high carcinogenic potential (69). However, recent studies have established a safe consumption limit, thus denying its genotoxic and carcinogenic potential (70).

Our study demonstrated a pattern similar to that practiced in other countries regarding the choice of implemented ingredients. Studies in other countries have shown the recurrent use of soy, wheat, and pea proteins as sensory and nutritional substitutes for meat, such as the use of fat sources such as vegetable oil, canola oil, and coconut fat (21, 22). Corn and potato starches were also used (21, 22).

The Brazilian market for meat substitutes is similar to the consumption pattern practiced by Brazilians. The present study found a higher frequency of red meat substitutes, followed by poultry, pigs, and fish. In Brazil, red meat's daily per capita consumption is 63.2 g, poultry is 36.5 g, fish is 23.4 g, and pork is 8.3 g (71). However, stratifying the data, the consumption of beef burgers is about 3 g daily *per capita*, and poultry meat burgers 0.9 g/day (71).

It is crucial to note that the Brazilian market for meat substitutes has positive growth projections, being valued at US$17 million in its first 3 months of launch (72). Despite this, surveys reveal a surge in the prevalence of vegetarianism in Brazil and an increase in the intention to purchase products classified as vegan (73). Therefore, it is crucial to invest in healthy alternatives that support the ethical and nutritional characteristics of plant-based vegetarian and vegan diets. Regarding the general characteristics of Brazilian vegan meat substitutes, potential benefits associated with their consumption may be noted, since their dietary fiber value is higher than the values found in animal protein counterparts. According to the WHO, a total of 25 g of dietary fiber should be consumed daily (62), and recent research on food consumption in Brazil points to a decrease in vegetable consumption in general, consequently leading to a reduction in the consumption of dietary fibers, which already tends to be reduced (61). However, despite the benefits resulting from the increased consumption of dietary fibers, it is noteworthy that these products are also incorporated with increased saturated fat and sodium levels, therefore, even though these are classified as plant-based products, these should still be interpreted as industrialized products, and their consumption should not be encouraged in a daily habit.

Despite being close in terms of nutritional value, different ingredients can influence human health in different ways, and, in the case of these products, the extensive use of artificial coloring and flavoring is necessary to obtain similar sensory aspects (22, 74). Therefore, further studies are needed to analyze the ingredients implemented in vegan meat substitutes.

One potential drawback of our investigation was the absence of laboratory chemical analysis to confirm the label information. According to the Brazilian legislation, nutritional labels can be based on food composition tables and present a discrepancy level of 20% (for more or less) between its actual chemical composition and that one described on the label (26). Thus, possible divergences related to Brazilian vegan meat substitutes real chemical composition may be present, as found in a study with other types of products (75).

## Conclusions

This study on the nutritional quality of vegan products commercialized in Brazil did not wholly confirm our hypothesis since most of them were similar to their animal counterparts on their labels' nutritional composition. Vegan meat substitutes presented similar energy and protein values, with few exceptions among samples, with vegan *canned fish* alternatives showing less protein than their counterparts. Naturally, vegan meat substitutes presented a higher concentration of carbohydrates and dietary fiber, given that plant-based ingredients also tend to present considerable amounts of these nutrients. Soy (mainly texturized soy protein) and gluten were most used as protein-sources in vegan products studied. Pea protein-derived ingredients were also used in the meat-free alternatives. Total and saturated fat was present in higher quantities among animal meat samples. Vegan meat analogs utilized vegetal fat as the main source. Regarding the sodium content, differences were found only in two groups, the vegan versions of *minced beef and fish cakes*, those with more sodium than their animal counterparts. Methylcellulose and natural aroma were the most implemented food additives.

Vegan meat substitutes did not differ from their respective animal counterparts. However, further studies aiming to research the chemical composition of these products with certified laboratory methods are needed. The Brazilian market for vegan meat substitutes is growing prosperously. Thus, there is a notorious demand for healthy alternatives contributing to adherence to vegetarian and vegan diets.

## Data Availability Statement

The original contributions presented in the study are included in the article/Supplementary Material, further inquiries can be directed to the corresponding author/s.

## Author Contributions

BR, RB, and RZ: conceptualization, methodology, investigation, data curation, and writing—original draft preparation. EN: software. RB and RZ: validation. BR, RB, RZ, and EN: formal analysis. RZ: resources. BR, RB, AR, and RZ: writing—review and editing. RB and AR: visualization. RZ, AV-M, AA-M, AR, and HH: supervision. AR, RZ, and HH: project administration. AR, RZ, AV-M, and AA-M: funding acquisition. All authors have read and agreed to the published version of the manuscript.

## Conflict of Interest

The authors declare that the research was conducted in the absence of any commercial or financial relationships that could be construed as a potential conflict of interest.

## Publisher's Note

All claims expressed in this article are solely those of the authors and do not necessarily represent those of their affiliated organizations, or those of the publisher, the editors and the reviewers. Any product that may be evaluated in this article, or claim that may be made by its manufacturer, is not guaranteed or endorsed by the publisher.
