# Supplementary material for: Are Vegan Alternatives to Meat Products Healthy? A Study on Nutrients and Main Ingredients of Products Commercialized in Brazil
Source: Front Public Health. 2022 May 27;10:900598. doi: 10.3389/fpubh.2022.900598 (PMC9235150; doi:10.3389/fpubh.2022.900598)
Supplement: Supplementary file 1 [file Table_1.DOCX]

**Table S1 - Full information of serving size, ingredient list and nutritional value of included meat substitutes**

| Classification | Ingredients | Serving Size | Energy Value (Kcal) | Carbohydrates (g) | Protein (g) | Total Fat (g) | Saturated Fat (g) | Dietary Fiber (g) | Sodium (mg) |
| --- | --- | --- | --- | --- | --- | --- | --- | --- | --- |
| Breaded Chicken | texturized soy protein, micronized protein, soy protein , gluten, soy sauce, garlic, salt, vegetable fat, panko flour, water and spices | 60 | 116 | 2,9 | 7,9 | 8,7 | 0 | 2,1 | 116,6 |
| Breaded Chicken | soy isolated fiber, isolated soy protein, water, sugar, soy oil, gluten, salt, panko flour,potato starch, wheat flour, spices, yeast extract | 100 | 198 | 14 | 15 | 9 | 0,5 | 6,5 | 768 |
| Breaded Chicken | water, bread crumbs, cottonseed oil, soy protein, vegetal fat, onion, gluten, cassava starch, riceflour, salt, cornflour | 130 | 449 | 31 | 12 | 28 | 5,6 | 5,5 | 791 |
| Breaded Chicken | water, bread crumbs, cottonseed oil, soy protein, vegetal fat, onion, gluten, cassava starch, riceflour, salt, cornflour | 130 | 291 | 29 | 17 | 12 | 1,3 | 4,8 | 801 |
| Breaded Chicken | texturized soy protein, isolated soy protein, water, soy oil, modified corn starch, sugar, salt, sorbitol, gluten, spices and natural aroma, white pepper, bread crumbs. | 40 | 127 | 4,9 | 6,5 | 9 | 1,4 | 1,7 | 286 |
| Breaded Chicken | texturized soy protein, isolated soy protein, gluten, modified starch, water, soy oil, salt, sorbitol and natural aroma. | 40 | 94 | 3,4 | 6,7 | 6 | 0,8 | 1,9 | 121 |
| Breaded Chicken | texturized soy protein, isolated soy protein, gluten, water, soy oil, sesame seed oil, sugar, salt, sorbitol, spices | 40 | 61 | 3 | 7,2 | 2,3 | 0,5 | 1,6 | 311 |
| Breaded Chicken | water, wheat flour , texturized pea protein, vegetal fat methylcellulose, modified starch, cornflour, starch, gluten, salt, garlic, onion | 130 | 330 | 32 | 13 | 15 | 2 | 1 | 750 |
| Breaded Chicken | water, wheat flour, texturized pea protein, vegetal fat, methylcellulose, modified starch, cornflour, starch, gluten, salt, garlic, onion | 80 | 210 | 32 | 8 | 9 | 1 | 1 | 460 |
| Breaded Chicken | water, soy protein, wheat flour , cornflour, oat, flaxseeds, sunflower oil, isolated soy protein, vegetal fiber, spices, natural aroma, salt, sugar, methylcellulose | 130 | 178 | 21,5 | 13,4 | 4,4 | 0,8 | 8,1 | 594 |
| Breaded Chicken | water, soy protein, wheat flour, cornflour, oat, flaxseeds, sunflower oil, isolated soy protein, vegetal fiber, spices, natural aroma, salt, sugar | 80 | 110 | 13 | 8,5 | 2,9 | 0,5 | 6,1 | 366 |
| Breaded Chicken | texturized pea protein, oil, salt, natural aroma | 100 | 188 | 24 | 13 | 4 | 0,7 | 5,1 | 376 |
| Breaded Chicken | texturized pea protein, oil, salt, bread crumbs, natural aroma | 100 | 188 | 24 | 13 | 4 | 0,7 | 5,1 | 376 |
| Breaded Chicken | texturized soy protein, micronized protein, soy protein , gluten, soy sauce, garlic, salt, vegetable fat, panko flour, water and spices. | 60 | 116 | 2,9 | 7,9 | 8,7 | 0 | 2,1 | 116,6 |
| Canned Fish | water, texturized pea protein, jackfruit, coconut fat, modified starch, 3% or less of onion, natural aroma, garlic, paprica e antioxidant ascorbic acid. | 90 | 146 | 12,1 | 11,9 | 5,6 | 4,2 | 4,2 | 337 |
| Canned Fish | potato, water, tomato, jackfruit, onion, texturized pea protein, olive oil, yellow bell pepper, red bell pepper, garlic, metyhlcelullose, modified starch, green olives, black olives, salt, epa, dha, ascorbic acid | 240 | 327 | 41 | 11 | 13 | 2,2 | 4,2 | 617 |
| Canned Fish | isolated soy protein, soy isolated fiber, water, gluten, soy oil, potato starch, sugar, salt, seaweed, spices, yeast extract, wheat flour. | 100 | 317 | 17 | 13 | 22 | 3,7 | 12 | 180 |
| Canned Fish | soy isolated fiber, isolated soy protein, water, soy oil, sugar, gluten, potato starch, salt, seaweed, spices, yeast extract. | 100 | 153 | 12 | 6 | 9 | 1,1 | 6,5 | 570 |
| Canned Fish | isolated soy protein, water, soy oil, sugar, gluten, salt, spices, yeast extract, xylose. | 100 | 138 | 19 | 2 | 6 | 3 | 2,8 | 723 |
| Canned Fish | water, pea protein, cottonseed oil, vegetal fat, gluten, salt, malt, onion, starch, sugar, iron, garlic, vitamin B12, methylcellulose, natural aroma, antioxidant ascorbic acid, beet coloring. | 300 | 304 | 19 | 21 | 16 | 2,2 | 5,5 | 777 |
| Canned Fish | water, bread crumbs, cottonseed oil, soy protein, vegetal fat, onion, gluten, cassava starch, riceflour, salt, cornflour | 130 | 440 | 31 | 12 | 28 | 5,6 | 5,5 | 721 |
| Canned Fish | water, texturized soy protein, pea protein e chickpea flour, olive oil , sugar, onion, salt, natural aroma and antioxidant ascorbic acid | 80 | 166 | 7,1 | 12 | 10 | 2,1 | 14 | 328 |
| Chicken Breast | water, texturized soy protein, isolated soy protein, pea protein and chickpea flour, vegetal fat, modified starch, onion, salt, dextrose, powdered beet, ascorbic acid | 80 | 168 | 11 | 8,2 | 10 | 9 | 0,7 | 392 |
| Chicken Breast | soy flour, soy sauce, garlic essential oil | 50 | 150 | 10 | 25 | 0 | 0 | 10 | 0,2 |
| Chicken Breast | soy flour, soy sauce, garlic essential oil | 50 | 150 | 10 | 25 | 0 | 0 | 10 | 0,2 |
| Chicken Breast | water, gluten, champignon, soy milk, garlic, salt, vegetable fat and spices. | 60 | 115 | 2,8 | 7,5 | 8,6 | 0 | 2 | 278 |
| Chicken Breast | water, texturized pea protein, modified starch, 2% or less of : onion, yeast extract, natural aroma, black pepper, iron e antioxidant ascorbic acid. | 30 | 34,8 | 2,9 | 3,8 | 0,2 | 0 | 1,6 | 97,8 |
| Chicken Breast | soy isolated fiber, isolated soy protein, water, soy oil, sugar, gluten, salt, yeast extract,spices, potato starch. | 100 | 153 | 12 | 6 | 9 | 1,1 | 6,5 | 571 |
| Chicken Breast | water, soy protein, pea protein, beans, sunflower oil, salt, flavorings, natural aroma | 80 | 94 | 3,1 | 17 | 1,6 | 0,4 | 2,6 | 560 |
| Chicken Breast | water, soy protein, pea protein, beans, sunflower oil, salt, flavorings, natural aroma | 80 | 94 | 3,1 | 17 | 1,6 | 0,4 | 2,6 | 560 |
| Chicken Breast | texturized soy protein, water, isolated soy protein, soy oil, gluten, salt. paprica, soy sauce, sorbitol, pepper, vegetal gum, spices. | 30 | 58 | 2,2 | 4,8 | 3,3 | 0,5 | 1,4 | 176 |
| Chicken Breast | texturized soy protein, soy protein , water, soy oil, cassava starch, gluten, carrageenan, salt, soy sauce, sorbitol, paprica, konjac, aroma and spices | 30 | 58 | 2,2 | 4,8 | 3,3 | 0,5 | 1,4 | 176 |
| Chicken Breast | water, (concentrated proteins of soy and pea), vegetable fat, maltodextrin de milho, salt, onion, black pepper, garlic, natural aroma, antioxidant ascorbic acid, vitamin B12 and iron. | 100 | 174 | 6 | 24 | 6 | 0 | 2 | 700 |
| Chicken Breast | water, (soy, pea and chickpea), vegetable fat, vegetal fiber, methylcellulose, maltodextrin, salt, yeast extract, natural aroma de frango, garlic, antioxidant ascorbic acid, vitamin B12 and iron. | 115 | 129 | 5,8 | 19 | 3,5 | 1,24 | 5 | 517 |
| Chicken Breast | water, (soy, pea and chickpea), vegetable fat, vegetal fiber, methylcellulose, cornflour, ginger, garlic, onion ,cilantro, white pepper, salt, natural aroma, antioxidant ascorbic acid, vitamin B12 and iron. | 120 | 138 | 8,4 | 18 | 3,6 | 1,3 | 5 | 504 |
| Chicken Hamburgers | soy flour, soy sauce, garlic essential oil | 50 | 150 | 10 | 25 | 0 | 0 | 10 | 0,2 |
| Chicken Hamburgers | water, (texturized soy protein, pea isolated protein), coconut oil, onion, garlic, pepper , iron, vitamins (B6 e B12), modified starch and methylcellulose, natural aroma, caramel color and antioxidant | 80 | 189 | 11 | 9,8 | 12 | 10 | 1 | 340 |
| Chicken Hamburgers | water, pea protein, cottonseed oil, vegetal fat, gluten, salt, malt, onion, starch, sugar, iron, garlic, vitamin B12, methylcellulose, natural aroma, antioxidant ascorbic acid, beet coloring. | 80 | 173 | 3,6 | 13 | 11 | 3 | 2,5 | 377 |
| Chicken Hamburgers | water, pea protein , orange pulp, sunflower oil, spices, yeast extract, vegetal fiber, salt, black pepper | 100 | 129 | 2,4 | 7 | 10 | 1,1 | 5 | 350 |
| Chicken Hamburgers | water, (soy, pea e chickpea), vegetal fiber, maltodextrin, methylcellulose, salt, natural aroma de frango, garlic, onion, white pepper, antioxidant ascorbic acid, vitamin B12 and iron. | 80 | 96 | 4,8 | 11 | 3,6 | 1,3 | 5 | 400 |
| Fish Cakes | texturized soy protein, micronized protein, soy protein , gluten, soy sauce, seaweed, garlic, salt, vegetable fat, panko flour, water and spices. | 60 | 116 | 3,6 | 7,5 | 9 | 1,1 | 2 | 280 |
| Fish Cakes | texturized soy protein, micronized protein, soy protein , gluten, soy sauce, seaweed, garlic, salt, vegetable fat, panko flour, water and spices. | 60 | 116 | 3,6 | 7,5 | 9 | 1,1 | 2 | 280 |
| Fish Cakes | water, potato, jackfruit, riceflour, texturized pea protein, olive oil, sunflower oil, cassava starch, methylcellulose, modified starch, cornflour, garlic, onion, black pepper, salt, dextrose, dha, epa, ascorbic acid | 100 | 206 | 28 | 8 | 8 | 1 | 1,5 | 406 |
| Fish Cakes | cashew fiber meat, tomato, onion, coconut milk, tomato extract, water flour, cilantro, flaxseed flour, garlic, salt, lime juice, apple vinegar, black pepper and annatto. | 40 | 41 | 7 | 1,6 | 0,7 | 0 | 3 | 199 |
| Fish Cakes | texturized soy protein, isolated soy protein, cassava starch, water, soy oil, gluten, carrots, sorbitol, salt, natural aroma and spices. | 30 | 82 | 3,5 | 4 | 5,7 | 0,9 | 2,3 | 172 |
| Hamburgers | water, texturized soy protein, pea protein e chickpea flour, vegetal fat, modified starch, onion, , salt, sugar, powdered beet, methylcellulose, natural aroma. | 80 | 200 | 10 | 9 | 13 | 5 | 0 | 520 |
| Hamburgers | texturized soy protein, water, soy oil, modified corn starch, sugar, salt, soy sauce, gluten, spices, black pepper moída, white pepper moída. | 50 | 118 | 2,8 | 7 | 9 | 1,3 | 2 | 344 |
| Hamburgers | texturized soy protein, soy extract, wheat flour, corn starch, water, soy oil, salt, sorbitol, soy sauce, oregano, spices. | 50 | 78 | 5,3 | 8,9 | 2,4 | 0,4 | 1,8 | 194 |
| Hamburgers | water, processed carrots, oat flakes, chickpea , quinoa, sesame seeds, green onion, onion, mustard, cassava starch, mustard, salt, garlic and spices. | 120 | 140 | 25 | 6,1 | 2,4 | 0,1 | 6,3 | 410 |
| Hamburgers | water, carrots, lentil, onion, flaked potato, cassava starch, spinach, palm oil, green onion, flaxseeds, salt, garlic, salt, sesame seeds e liquid smoke. | 60 | 128 | 19 | 5,1 | 2,8 | 1,4 | 1,9 | 284 |
| Hamburgers | water, chickpea, beans, olive oil, pataua oil, cilantro, cumin, curry, black pepper, sodium bicarbonate, salt, sesame seeds, green onion, parsley, mint and green onion. | 113 | 253 | 42 | 17 | 2,1 | 0 | 10 | 308 |
| Hamburgers | water, (soy concentrated protein e pea concentrated protein), bamboo fiber, onion, vegetal fat, salt, beet, yeast extract, black pepper, garlic, methylcellulose, natural aroma, caramel color, antioxidant ascorbic acid, vitamin B12 and iron. | 80 | 90 | 4,8 | 11 | 3 | 1,1 | 4 | 450 |
| Hamburgers | water, texturized soy protein, pea isolated protein, coconut oil, onion, salt, garlic, , powdered beet, iron, vitamins (B6 and B12), modified starch e methylcellulose, natural aroma e antioxidant. | 80 | 187 | 10 | 9,8 | 12 | 10 | 1 | 330 |
| Hamburgers | water pea concentrated protein, pea texturized protein, vegetal fiber, sunflower oil, coconut oil, pea isolated protein, yeast extract, spices, vegan beef, onion powder, beet concentrate, garlic powder, salt, smoke arome, spices, minerals: iron e zinc, vitamins: A, B9 and B12. | 113,5 | 158 | 8,7 | 15 | 7,9 | 4,3 | 5 | 459 |
| Hamburgers | water, pea protein, cottonseed oil, vegetal fat, gluten, salt, malt, onion, starch, sugar, iron, garlic, vitamin B12, methylcellulose, natural aroma, antioxidant ascorbic acid, beet coloring. | 100 | 206 | 4,1 | 13 | 14 | 3 | 3,2 | 495 |
| Hamburgers | water, pea protein, coconut oil, sunflower oil, bamboo fiber, cacao powder, rice protein, salt, powdered beet, chia seeds , spinach , minerals (iron and zinc), vitamin A, vitamin B9, vitamin B12, methylcellulose and natural aroma | 100 | 420 | 4,4 | 28 | 32 | 18,4 | 11,6 | 882 |
| Hamburgers | water, pea protein, soy protein, vegetal fat, cashew fiber meat, onion, garlic, salt, pepper, açai extract, powdered beet, natural aroma and methylcellulose. | 115 | 209 | 5,3 | 20 | 12 | 5,7 | 9,1 | 295 |
| Hamburgers | water, texturized soy protein, soy protein ,micronized protein, vegetable fat, gluten, salt, spices. | 60 | 114 | 4,5 | 7,6 | 7,3 | 0 | 3,5 | 112,6 |
| Hamburgers | water, gluten, coconut oil, soy protein, sunflower oil, onion, beet, potatofiber, herbs e spices, salt, garlic, tomato, maltodextrin, iron, vitamin B12, flavorings: natural aroma, methylcellulose. | 80 | 170 | 4,6 | 12 | 12 | 6,4 | 0 | 4,1 |
| Hamburgers | water, texturized pea protein, coconut fat, modified starch, onion, yeast extract, powdered beet, natural aroma, black pepper, garlic, iron and antioxidant ascorbic acid. | 60 | 134 | 5,9 | 7,3 | 9 | 7,4 | 2,8 | 258 |
| Hamburgers | water, texturized soy protein, carrots in natura, corn, bread crumbs, wheat flour, onion in natura, pea, palm oil , corn starch, salt, salt, garlic and spices. | 134 | 237 | 29 | 16 | 6,9 | 3,1 | 1,9 | 604 |
| Hamburgers | water, texturized soy protein, wheat flour, bread crumbs, onion in natura, palm oil , salt, corn starch, salt, garlic and spices. | 60 | 115 | 13 | 8,9 | 3,3 | 1,5 | 0,4 | 244 |
| Hamburgers | water, texturized soy protein, wheat flour, bread crumbs, vegetal fat, sunflower oil, corn starch, salt, salt, spices. citric acid. caramel color, anatto natural coloring. | 60 | 118 | 11 | 6 | 5,7 | 2,7 | 0 | 336 |
| Hamburgers | water, texturized soy protein, wheat flour, bread crumbs, vegetal fat, sunflower oil, corn starch, salt, salt, spices. citric acid. caramel color, anatto natural coloring. | 60 | 118 | 11 | 6 | 5,7 | 2,7 | 0 | 336 |
| Hamburgers | water, texturized soy protein, pea protein e chickpea flour, vegetal fat, modified starch, onion, , salt, sugar, powdered beet, methylcellulose, natural aroma and antioxidant | 80 | 197 | 10 | 11 | 13 | 4,2 | 2,8 | 476 |
| Hamburgers | water, white quinoa, black quinoa, red quinoa, oat flakes, carrots, zucchini, onion, black tucupi, apple vinegar, olive oil, salt, nutmeg, black pepper and cilantro. | 115 | 309 | 45 | 7 | 11 | 0 | 4,2 | 273 |
| Hamburgers | sweet potato, oat, carrots, white quinoa, red quinoa, olive oil, lime, salt, onion, garlic, curry, oregano, basil, cilantro, cumin and mint | 90 | 127 | 23 | 3,1 | 3,2 | 0,4 | 2,9 | 376 |
| Hamburgers | soy flour, soy sauce, garlic essential oil and onion | 50 | 150 | 10 | 25 | 0 | 0 | 10 | 0,2 |
| Hamburgers | beans, oat, olive oil , golden flax seeds, chia seeds, salt, onion, garlic, turmeric, annatto, liquid smoke , cilantro, salt, green onion, cumin, oregano and bay. | 90 | 120 | 17 | 5,4 | 3,7 | 0,5 | 7 | 386 |
| Hamburgers | beans, oat, beet, tomato extract, olive oil , golden flax seeds, chia seeds, salt, white wine vinegar, onion, garlic, smoked paprica, salt, cayene pepper, chipotle, chili, red pepper, black pepper, liquid smoke , cumin and cilantro. | 90 | 171 | 28 | 3,4 | 0,4 | 0 | 8,2 | 387 |
| Hamburgers | soy isolated fiber, isolated soy protein, water, sugar, soy oil, gluten, salt, yeast extract,potato starch, spices. | 100 | 196 | 9,7 | 19 | 9,2 | 1,5 | 6,8 | 575 |
| Hamburgers | soy isolated fiber, isolated soy protein, water, soy oil, sugar, chestnut, salt, celery, gluten, potato starch, carrots, yeast extract, | 63 | 146 | 7 | 12,3 | 7,6 | 1,3 | 3,8 | 438 |
| Hamburgers | gluten,soy oil, wheat fiber, wheat flour , texturized soy protein, garlic, onion, salt, spices, maltodextrin, sugar, yeast extract, caramel color, chia seeds and natural aroma. | 70 | 80 | 4,4 | 8,2 | 3,3 | 0,8 | 3,1 | 257,6 |
| Hamburgers | chickpea, water, flaked potato, onion, palm oil, chickpea flour, cassava starch, chickpea flour, salt, green onion, flaxseeds, salt and spices. | 67 | 301 | 40 | 8,9 | 12 | 4,8 | 6,3 | 511 |
| Hamburgers | chickpea, carrots, oat, lime juice, olive oil , salt, golden flax seeds, chia seeds, sesame seeds, zathar, onion, garlic, smoked paprica, black pepper, manjerona, salt, oregano, cumin and mint | 90 | 192 | 26 | 7,7 | 6,8 | 1 | 6,8 | 371 |
| Hamburgers | lentil, oat, girassol, soy sauce, olive oil , lime juice, golden flax seeds, chia seeds, salt, onion, garlic, smoked paprica, sesame seed oil, cilantro, saltm ginger, black pepper | 90 | 210 | 29 | 10 | 3 | 0,6 | 8 | 400 |
| Hamburgers | texturized soy protein, wheat flour, gluten, water, bread crumbs, shimeji, shitake, vegetable fat, salt, garlic | 60 | 110 | 8,9 | 4,3 | 4 | 0 | 4,4 | 150 |
| Hamburgers | soy protein, pea, quinoa, garlic, onion, vegetable fat, spices, salt e flavoring | 50 | 62 | 1 | 10 | 0 | 0 | 0 | 188 |
| Hamburgers | texturized soy protein, oat, flaxseed flour, onion powder, sunflower oil, quinoa flakes, salt, cassava starch, garlic, carob powder, powdered beet, soluble fiber and natural aroma | 80 | 266 | 26 | 27 | 6 | 0,8 | 11 | 315 |
| Hamburgers | texturized soy protein, oat, flaxseed flour , onion, sunflower oil, quinoa flakes, salt, cassava starch, herbs (parsley, green onion), garlic, carob powder, powdered beet, soluble fiber and aroma. | 80 | 266 | 26 | 27 | 6 | 0,8 | 11 | 315 |
| Hams | soy isolated fiber, isolated soy protein, water, sugar, soy oil, gluten, salt, yeast extract, potato starch, spices natural. | 40 | 78 | 3,9 | 7,4 | 3,7 | 0,6 | 2,7 | 230 |
| Hams | water, potato starch, taro starch, corn starch,cassava starch, modified starch , citric acid, sunflower oil | 60 | 185 | 31 | 1,1 | 6,3 | 0,7 | 0 | 778 |
| Hams | texturized soy protein, water, isolated soy protein, soy oil, gluten, salt, paprica, soy sauce, sorbitol, pepper, vegetal gum, spices. | 20 | 34 | 0,2 | 3,4 | 2,2 | 0,2 | 1,5 | 227 |
| Hams | texturized soy protein, soy extract, cassava starch, corn starch, wheat flour, gluten, water, soy oil, salt, sugar, sorbitol, soy sauce, natural aroma. | 40 | 75 | 3,6 | 7,6 | 3,3 | 0,5 | 3,1 | 395 |
| Hams | texturized soy protein, soy protein , cassava starch, gluten, carrageenan, water, soy oil, salt, soy sauce, sorbitol, aroma and spices. | 20 | 35 | 0,4 | 3,9 | 2 | 0,2 | 1,53 | 226 |
| Hams | Soy, pea and chickpea, vegetable fat, vegetal fiber, salt yeast extract, sugar, extrato de malt, natural aroma , antioxidant ascorbic acid. | 100 | 476 | 14 | 42 | 28 | 0 | 4 | 600 |
| Meatballs | water, texturized soy protein, isolated soy protein, pea protein e chickpea flour, vegetal fat, modified starch, onion, salt, dextrose, powdered beet, ascorbic acid | 80 | 168 | 11 | 8,2 | 10 | 9 | 0,7 | 392 |
| Meatballs | soy flour, soy sauce, garlic essential oil,onion | 50 | 150 | 10 | 25 | 0 | 0 | 10 | 0,2 |
| Meatballs | soy flour, soy sauce, garlic essential oil e onion | 50 | 150 | 10 | 25 | 0 | 0 | 10 | 0,2 |
| Meatballs | soy protein, pea, quinoa, garlic, onion, vegetable fat, spices, salt e flavoring | 50 | 62 | 1 | 10 | 0 | 0 | 0 | 188 |
| Meatballs | soy protein, pea, quinoa, garlic, onion, vegetable fat, spices, salt e flavoring | 50 | 62 | 1 | 10 | 0 | 0 | 0 | 188 |
| Meatballs | water, texturized soy protein, modified corn starch, gluten, soy sauce, onion, garlic, salt, vegetable fat and spices | 60 | 115 | 2,8 | 7,5 | 8,6 | 0 | 2 | 278 |
| Meatballs | water, texturized soy protein, modified corn starch, gluten, onion, soy sauce, onion, garlic, salt, vegetable fat e spices | 60 | 115 | 3,1 | 7,5 | 8,6 | 0 | 2 | 278 |
| Meatballs | water, pea protein, cottonseed oil, vegetal fat, gluten, salt, malt, onion, starch, sugar, iron, garlic, vitamin B12, methylcellulose, natural aroma, antioxidant ascorbic acid, beet coloring. | 350 | 121 | 9,3 | 9,3 | 4,8 | 0,7 | 0,8 | 946 |
| Meatballs | texturized soy protein, water, soy protein, soy oil, gluten, salt, corn starch, carrots, carrageenan, soy sauce, sorbitol and spices. | 40 | 88 | 6 | 6,3 | 4,3 | 0,6 | 1,5 | 283 |
| Meatballs | texturized soy protein, water, soy oil, salt, sugar, gluten, soy sauce, sorbitol, black pepper, brazilian spices, spices and natural aroma. | 80 | 129 | 12 | 11 | 4,3 | 0,7 | 4 | 398 |
| Meatballs | water, soy protein, palm oil, corn starch, sunflower oil, spices, bamboo fiber, caramel color, natural aroma, and methylcellulose. | 100 | 203 | 13 | 9,2 | 13 | 4,3 | 5,2 | 514 |
| Meatballs | gluten, texturized soy protein, soy flour, soy oil, salt, spices and caramel color. | 40 | 64 | 2,7 | 7,8 | 2,4 | 0,4 | 1,3 | 316 |
| Meatballs | water, texturized soy protien, potato starch, onion, garlic, salt, spices, maltodextrin, sugar, yeast extract and caramel color. | 50 | 26 | 0,5 | 5 | 0 | 0 | 3,8 | 338 |
| Meatballs | water, texturized soy protein, potato starch, onion, garlic, spices, salt, maltodextrin, sugar, yeast extract and caramel color. | 50 | 22 | 0,5 | 5,3 | 0 | 0 | 2,5 | 323 |
| Meatballs | water , texturized soy protein, isolated soy protein, gluten, vegetable fat, tomato juice, modified starch, salt, sugar, maltodextrin, yeast extract, onion, garlic, spices and caramel color | 50 | 53 | 0 | 5,4 | 3,5 | 0,4 | 2,8 | 203 |
| Meatballs | texturized soy protein, isolated soy protein, , gluten, soy oil, modified starch, salt, onion, garlic, tomato pulp, sugar, maltodextrin, yeast extract, spices (black pepper, garlic, onion) and caramel color. | 50 | 63 | 0,2 | 6,3 | 4,1 | 0,4 | 3,6 | 316 |
| Meatballs | water, (soy, pea, chickpea), vegetable fat, vegetal fiber, spicesm salt, tomato pulp, yeast extract, sugar, vinegar, onion, garlic, extrato de malt, natural aroma, antioxidant ascorbic acid, vitamin B12 and iron. | 100 | 144 | 5 | 22 | 4 | 0,52 | 5 | 420 |
| Meatballs | water, (soy, pea, chickpea), vegetable fat, vegetal fiber, spices , salt, yeast extract, sugar, onion, garlic, extrato de malt, natural aroma , antioxidant ascorbic acid. | 100 | 119 | 5 | 18 | 3 | 0,4 | 5 | 400 |
| Minced Beef | water, texturized soy protein, pea protein e chickpea flour, vegetal fat, modified starch, onion, salt, sugar, powdered beet, methylcellulose, natural aroma and antioxidant | 80 | 168 | 11 | 8,2 | 10 | 9 | 0,7 | 392 |
| Minced Beef | water, soy protein, wheat, cottonseed oil, onion, salt, gluten, garlic, salt, iron, vitamin B12 | 100 | 166 | 10 | 13 | 11 | 0 | 4,3 | 686 |
| Minced Beef | water, pea protein, cottonseed oil, vegetal fat, gluten, salt, malt, onion, starch, sugar, iron, garlic, vitamin B12, methylcellulose, natural aroma , antioxidant ascorbic acid, beet coloring. | 100 | 166 | 10 | 13 | 11 | 0 | 4,3 | 686 |
| Minced Beef | water, texturized soy protein, bread crumbs, onion, palm oil , salt, garlic, spices and spices. | 75 | 178 | 33 | 4,3 | 3,4 | 1,3 | 3,6 | 494 |
| Minced Beef | water, soy protein, sunflower oil, corn starch, palm oil, spices , bamboo fiber, spices, natural aroma, caramel color and methylcellulose. | 80 | 158 | 9,3 | 7,8 | 9,9 | 2,3 | 4,1 | 615 |
| Minced Beef | gluten, , texturized soy protein, soy flour, vegetal fat , salt, sugar, maltodextrin, yeast extract, spices (black pepper, garlic, onion) and caramel color. | 50 | 77 | 2,1 | 13 | 1,9 | 0,3 | 1,5 | 253 |
| Minced Beef | water, (soy, pea, chickpea), vegetable fat, vegetal fiber, spices (natural aroma de bovino), salt, yeast extract, sugar, onion, garlic, antioxidant ascorbic acid, beet, extrato de malt, vitamin B12 e iron. | 100 | 188 | 6 | 14 | 12 | 3,6 | 4,5 | 450 |
| Minced Beef | soy isolated fiber, isolated soy protein, water, soy oil, sugar, carrots, salt, celery, gluten, chestnut, potato starch, spices, yeast extract. | 100 | 234 | 12 | 19 | 12 | 2 | 17 | 655 |
| Minced Beef | water, pea protein, soy protein, vegetal fat, cashew fiber meat, onion, garlic, salt, pepper, açai extract, powdered beet, natural aroma and methylcellulose. | 80 | 146 | 3,7 | 14 | 8,6 | 4 | 6,4 | 205 |
| Sausages | water, texturized soy protein, isolated soy protein, pea protein e chickpea flour, vegetal fat, modified starch, salt, powdered beet, natural aroma, sugar, onion, garlic, white pepper, methylcellulose, carrageenan, prepared condiment, e antioxidant ascorbic acid. | 50 | 139 | 7,7 | 5,7 | 9,5 | 3,9 | 0,2 | 294 |
| Sausages | water, texturized soy protein, soy protein , modified corn starch, cassava starch, vegetable fat, gluten, salt, spices, artificial coloring | 35 | 78 | 6 | 4 | 3,6 | 0 | 0,9 | 230 |
| Sausages | chickpea, texturized soy protein, soy protein, micronized protein, gluten, vegetable fat, salt, spices, anatto natural coloring | 35 | 78 | 6 | 4 | 3,6 | 0,5 | 0,9 | 230 |
| Sausages | soy isolated fiber, water, gluten, soy oil, sugar, potato starch, salt, spices, yeast extract, red rice yeast powder. | 65 | 156 | 8 | 11 | 9 | 1,5 | 13 | 463 |
| Sausages | water, pea protein, cottonseed oil, vegetal fat, gluten, salt, malt, onion, starch, sugar, iron, garlic, vitamin B12, methylcellulose, natural aroma, antioxidant ascorbic acid, beet coloring. | 80 | 115 | 3,6 | 9,7 | 6,7 | 0,4 | 0,5 | 255 |
| Sausages | texturized soy protein, water, isolated soy protein, soy oil, gluten, salt, paprica, soy sauce, sorbitol, pepper, vegeta lgum, konjac and spices. | 30 | 44 | 0,5 | 5,1 | 2,4 | 0,4 | 1,2 | 171 |
| Sausages | texturized soy protein, isolated soy protein, cassava starch, water, soy oil, gluten, carrageenan, sorbitol, salt, paprica, soy sauce, natural aroma and spices. | 30 | 58 | 1,7 | 5,1 | 3,4 | 0,5 | 1,2 | 197 |
| Sausages | texturized soy protein, isolated soy protein, cassava starch , gluten, vegetal gum, water, soy oil, salt, sugar, paprica, soy sauce, sorbitol, natural aroma and spices. | 50 | 93 | 1,9 | 8,1 | 5,9 | 0,5 | 1,9 | 294 |
| Sausages | texturized soy protein, water, isolated soy protein, soy oil, gluten, salt, paprica, soy sauce, sorbitol, pepper, vegetal gum, spices. | 30 | 58 | 4,3 | 2,4 | 0,6 | 0,5 | 1,3 | 161 |
| Sausages | texturized soy protein, isolated soy protein, cassava starch , gluten, vegetal gum, water, soy oil, salt, paprica, soy sauce, sorbitol, aroma and spices. | 50 | 96 | 1,8 | 8,8 | 5,9 | 0,4 | 2 | 308 |
| Sausages | gluten, texturized soy protein, soy flour, soy oil, vegetal fiber, salt, sugar, maltodextrin, spices | 40 | 60 | 1,4 | 7,6 | 2,7 | 0,4 | 1,6 | 244 |
| Sausages | gluten, vegetal fiber, soy oil, wheat fiber, wheat flour, soy flour, corn starch, black pepper, salt, sugar, maltodextrin, spices (garlic, cilantro , onion, black pepper) yeast extract, and natural aroma . | 40 | 43 | 1,8 | 4,9 | 1,9 | 0,5 | 2,5 | 143 |

**Table S2 - Full information of serving size and nutritional values of included animal protein counterparts**

| Classification | Sample | Serving Size (g) | Energy (Kcal) | Carbohydrates (g) | Protein (g) | Total Fat (g) | Saturated Fat (g) | Dietary Fiber (g) | Sodium (mg) |
| --- | --- | --- | --- | --- | --- | --- | --- | --- | --- |
| Hamburgers | 1 | 80 | 225 | 3 | 15 | 17.5 | 5.1 | 1.3 | 405 |
| Hamburgers | 2 | 80 | 155 | 2.1 | 12 | 11 | 3.8 | 0 | 520 |
| Hamburgers | 3 | 80 | 168 | 2.4 | 13 | 12 | 6 | 0 | 503 |
| Minced Beef | 1 | 100 | 241 | 0 | 24 | 15 | 6 | 0 | 73 |
| Minced Beef | 2 | 100 | 254 | 0 | 22 | 18 | 8 | 0 | 81 |
| Minced Beef | 3 | 100 | 248 | 0 | 23 | 14 | 6 | 0 | 74 |
| Meatballs | 1 | 80 | 142 | 8 | 9.6 | 7.9 | 3.5 | 0 | 598 |
| Meatballs | 2 | 80 | 200 | 2.5 | 10 | 12 | 5.9 | 0.5 | 474 |
| Meatballs | 3 | 80 | 138 | 7.7 | 12 | 6.6 | 3.3 | 0.5 | 486 |
| Breaded Chicken | 1 | 130 | 242 | 17 | 20 | 10 | 4.1 | 2.1 | 624 |
| Breaded Chicken | 2 | 130 | 284 | 18 | 17 | 16 | 5 | 1.7 | 809 |
| Breaded Chicken | 3 | 130 | 334 | 24 | 15 | 20 | 6.8 | 2.3 | 476 |
| Chicken Hamburgers | 1 | 80 | 201 | 2.6 | 15 | 13.8 | 4.6 | 1 | 221 |
| Chicken Hamburgers | 2 | 80 | 133 | 2.4 | 12 | 8.4 | 2.5 | 0.8 | 606 |
| Chicken Hamburgers | 3 | 80 | 153 | 2.4 | 13 | 10 | 2.9 | 0 | 484 |
| Chicken Breast | 1 | 100 | 165 | 0 | 31 | 3.6 | 1 | 0 | 74 |
| Chicken Breast | 2 | 100 | 165 | 0 | 31 | 3.6 | 1 | 0 | 74 |
| Chicken Breast | 3 | 100 | 160 | 0 | 30 | 3.4 | 1 | 0 | 74 |
| Canned Fish | 1 | 60 | 81 | 0 | 17 | 1.6 | 0.5 | 0 | 248 |
| Canned Fish | 2 | 60 | 114 | 0 | 14 | 6.5 | 1.3 | 0 | 330 |
| Canned Fish | 3 | 100 | 153 | 0 | 23 | 6.7 | 3.3 | 0 | 283 |
| Fish Cakes | 1 | 90 | 102 | 17 | 6.8 | 6.8 | 0.4 | 0.5 | 175 |
| Fish Cakes | 2 | 63 | 125 | 0 | 19 | 4.8 | 2.1 | 0 | 50 |
| Fish Cakes | 3 | 100 | 181 | 9.8 | 11 | 11 | 2.5 | 7.2 | 390 |
| Sausages | 1 | 50 | 116 | 1.5 | 6.8 | 8.5 | 3 | 0 | 399 |
| Sausages | 2 | 50 | 133 | 1.7 | 6.8 | 11 | 3.7 | 0 | 560 |
| Sausages | 3 | 50 | 160 | 0.8 | 8.8 | 14 | 4.5 | 0 | 725 |
| Hams | 1 | 40 | 35 | 0.6 | 6.1 | 0.9 | 0.3 | 0 | 267 |
| Hams | 2 | 40 | 35 | 0 | 6.4 | 0.9 | 0.3 | 0 | 412 |
| Hams | 3 | 40 | 121 | 3.1 | 4.8 | 9.9 | 3.4 | 0 | 600 |
